# An examination of sociodemographic and clinical factors influencing help-seeking attitudes and behaviors among adolescents with mental health problems

**DOI:** 10.1007/s00787-024-02568-7

**Published:** 2024-08-27

**Authors:** Marialuisa Cavelti, Noemi Anne Ruppen, Silvano Sele, Markus Moessner, Stephanie Bauer, Katja Becker, Jennifer Krämer, Heike Eschenbeck, Christine Rummel-Kluge, Rainer Thomasius, Silke Diestelkamp, Vera Gillé, Sabrina Baldofski, Julian Koenig, the ProHEAD-Consortium, Michael Kaess

**Affiliations:** 1 University Hospital of Child and Adolescent Psychiatry and Psychotherapy, University of Bern, Bolligenstrasse 111, 3000 Bern 60, Switzerland; 2 Department of Child and Adolescent Psychiatry, Centre for Psychosocial Medicine, University of Heidelberg, Heidelberg, Germany; 3 Centre for Psychotherapy Research, Centre for Psychosocial Medicine, University of Heidelberg, Heidelberg, Germany; 4 Department of Psychiatry and Psychotherapy, Medical Faculty, Leipzig University, Leipzig, Germany; 5 Department for Child and Adolescent Psychiatry, Psychosomatics and Psychotherapy, Faculty of Medicine, University of Marburg, Marburg, Germany; 6 Department of Child and Adolescent Psychiatry, Psychosomatics and Psychotherapy, Faculty of Medicine, University of Cologne, University Hospital Cologne, Cologne, Germany; 7 German Center for Addiction Research in Childhood and Adolescence, University Hospital Hamburg-Eppendorf, Hamburg, Germany; 8 Department of Educational Psychology and Health Psychology, University of Education Schwäbisch Gmünd, Schwäbisch Gmünd, Germany

**Keywords:** Help-seeking, Adolescents, Mental health problems, Predictors

## Abstract

This study investigated sociodemographic and clinical factors influencing help-seeking attitudes and behavior among adolescents with mental health problems. As part of the ProHEAD (“Promoting Help-seeking using E-technology for ADolescents”) consortium a school-based, online assessment of sociodemographic information, psychopathology, risk-taking and self-harming behavior, help-seeking attitudes and behavior, and barriers to help-seeking was conducted in adolescents aged ≥ 12 years recruited from randomly selected schools in five regions of Germany. Linear regression analyses with the LMG formula were performed to explore predictors of help-seeking attitudes and behavior and assess their relative importance. Nine thousand five hundred and nine participants (95.5%) completed the online assessment (mean age: 15.1 years, 58.6% female). In total, 1606 participants (16.9%) showed relevant mental health problems (e.g., depressive and eating disorder symptoms, alcohol problems, and thoughts of self-harming behavior). Among them, 895 (55.7%) reported having sought help (lifetime), with higher rates for informal (n = 842, 52.4%) compared to professional (*n* = 380, 23.7%) sources. High help-seeking propensity emerged as the most important factor contributing to professional help-seeking, followed by elevated levels of psychopathology and perceived barriers, with sociodemographic factors being less impactful. Psychopathological severity also outweighed sociodemographic factors in predicting negative help-seeking attitudes. These findings indicate that attitudes towards seeking professional help, perceived barriers, and psychopathology severity critically influence limited adolescent help-seeking behavior. This emphasizes the need for initiatives that promote help-seeking, reduce negative attitudes, and address structural barriers in adolescent mental health care.

**Supplementary Information:**

The online version contains supplementary material available at 10.1007/s00787-024-02568-7.

## Introduction

Nearly half of all mental disorders begin before the age of 18, and 62% manifest before the age of 25, with a peak age of onset for all mental disorders at 14.5 years [1]. In Europe, almost one in five children and adolescents suffer from a mental disorder [2]. Developing mental health issues before 14 years of age increases the risk of adult mental disorder [3]. Mental disorders are the leading cause of Years Lived with Disability (YLDs), and self-harm is a prominent cause of Years of Life Lost (YLLs) among young people in most European countries [4], highlighting the negative long-term impact of mental disorders at a young age on both individuals and societies.

Despite the high prevalence and significant impact of mental disorders in young people, a large proportion of them do not seek help. Help-seeking is recognized as an *“adaptive process that is the attempt to obtain external assistance to deal with a mental health concern”* that can be professional (e.g., mental health professionals, primary health care providers, teachers) or informal (e.g., friends, parents) [5]. In a representative school-based study across 11 European countries, 61.9% of adolescents aged 13–17 years were at risk for a mental disorder or risk behavior [6], but only 10% sought professional treatment within one year. Additionally, 4.2% reported current suicidality, yet the majority did not seek professional help within a year [7]. The COVID-19 pandemic has further exacerbated the delay and reduction in help-seeking for mental health problems [8].

Recent systematic reviews of qualitative and quantitative studies exploring barriers and facilitators of help-seeking for mental health problems from the perspective of the adolescents and their parents have identified various factors influencing help-seeking behavior that lie with the adolescents/families, the society, or the healthcare system [9–11]. These barriers include limited knowledge about mental health and available services, prior negative experiences with mental health professionals, negative attitudes towards mental health problems and help-seeking, stigmatization, preference for self-help or informal support, confidentiality concerns, service and indirect costs (e.g., travel costs, loss of wages), logistical barriers (e.g., long travel distances, limited availability of parents), and limited access to professional help (e.g., waiting times, difficulty in getting a referral, inflexible appointment systems) [10, 11]. However, the current evidence is constrained by selective samples (e.g., adolescents/families who accessed services), the lack of evaluated measures of mental health problems and of facilitators/barriers for help-seeking, the focus on help-seeking attitudes or intentions rather than actual help-seeking behavior, and the insufficient examination of the relative importance of different factors influencing help-seeking behavior.

To address these gaps, the current study had three main objectives. Firstly, to examine in a large school-based sample of adolescents the rates of professional and informal help-seeking for mental health problems. Secondly, to explore various sociodemographic and clinical factors associated with attitudes towards seeking assistance and actual help-seeking behavior and assess their relative importance. According to the theory of planned behavior, attitude is not truly a measure of help-seeking in the sense of an active coping attempt but influences observable behavior [5]. Thirdly, to investigate potential differences in sociodemographic and clinical factors influencing help-seeking attitudes and behavior between adolescents who exceeded a pre-defined threshold for relevant mental health problems (i.e., “the clinical group”) and those who did not (i.e., “the non-clinical group”).

## Methods

### Sample and procedure

The present study is a data analysis of baseline screening data collected within the ProHEAD (“Promoting Help-seeking using E-technology for ADolescents”) consortium. The consortium aims at (1) improving help-seeking behavior in young people who experience relevant mental health problems; (2) improving the selective prevention of common disorders in those who are at risk; and (3) strengthening resources to counteract the development of mental disorders in young people [12]. The consortium conducted longitudinal, school-based, online assessments of mental health problems in adolescents between 12 and 25 years old. After the completion of the initial screening assessment, participants were allocated to one of five online randomized controlled trials based on their screening results, followed by two annual follow-up assessments. The baseline screening was conducted at randomly selected schools in five regions of Germany (i.e., Heidelberg, Hamburg, Leipzig, Schwäbisch Gmünd, Marburg). The randomized selection was stratified by regional district and school-type to ensure the selection of a representative sample of schools within each recruitment area. Inclusion criteria were sufficient German skills and internet access. Eligible adolescents and their legal guardians were asked to provide written informed consent before participating. The study protocol was approved by the Ethics Committee of the Medical Faculty at the University of Heidelberg (S-086/2018; leading study site) as well as the respective institutional review boards of the additional study sites. For the present study, the data of the initial screening were used.

### Instruments

#### Sociodemographic information

Sociodemographic information included age, gender [f/m], center [Heidelberg, Hamburg, Leipzig, Schwäbisch Gmünd or Marburg], school type [Gymnasium, Realschule, Haupt- und Werkrealschulen, Gemeinschaftsschulen (Oberschule, Stadtteilschule), Berufsschule, other], and migration status (no [0], unknown [2] or yes [1] , meaning that the hild or one of the parent was *not* born in Germany). The socioeconomic status (SES) was assessed using the *Family Affluence Scale (FAS)* [13]. The FAS is a short four-item measure, with a total score ranging between 0 and 2 indicating low, between 3 and 5 medium, and between 6 and 9 high SES. The Laucht-Index [14] was applied to assess psychosocial adversity (e.g. parental unemployment) that may pose a risk on the development of a child. It comprises 10 items and distinguishes no risk [0], low risk [1, 2] and high risk [> 2]. For the current analysis, the mean scores of the FAS and the Laucht-Index were used.

#### Psychopathology

The *KIDSCREEN-10* was used to assess health-related quality of life [15]. It contains ten items with a score ranging from 0 [never/not at all/excellent] to 4 [very much/always/bad]. A higher total score reflects greater health-related quality of life. Furthermore, the *Strengths and Difficulties Questionnaire (SDQ)* [16] was applied to assess mental health problems in the previous six months. It is a 25-item measure, with each item being scored from 0 [not applicable] to 2 [clearly applicable]. A greater total score indicates more psychological problems. An extended version of the *Patient Health Questionnaire-9 modified for adolescents (PHQ-A)* [17] was used as a severity measure for depressive symptoms within the past two weeks. This questionnaire consists of ten items scored from 0 [not at all/no] to 3 [almost daily/extremely] as well as three items scored as 0 [no] or 1 [yes]. A greater total score means more severe symptoms of depression.

Features of eating disorder pathology in the previous four weeks were assessed by using the *Eating Disorder Examination-Questionnaire for Children (ChEDE-Q)* [18]. The ChEDE-Q is a 22-item measure scored from 0 [no day/never/not at all] to 6 [everyday/every time/clearly uncomfortable]. The total mean score was used as an indicator of severity of eating disorder symptoms.

A brief screening for personality disorders was performed using the *Self-Rated Standardized Assessment of Personality - Abbreviated Scale (SAPAS)* [19]. This questionnaire consists of eight items with dichotomous answer options [no = 0/yes = 1], with a greater total score reflecting more severe personality pathology.

#### Risk-taking behavior and self-harm

The *Alcohol Use Disorders Identification Test (AUDIT)* [20], which is composed of ten items, was applied to detect signs of alcohol use disorders in the previous 12 months. Its items are coded from 0 [never/1–2 glasses/no] to 4 [four times a week or more/ten or more/yes in the last year]. A greater total score indicates more severe alcohol misuse.

Suicidal ideation and attempts were assessed using the *Paykel Scale* [21]. Two items measured suicidal thoughts and plans in the past 12 months, rated on a scale from 0 [never] to 5 [always]. In contrast, suicidal thoughts and plans in the last two weeks were assessed as either present [1] or absent [0]. The occurrence of suicidal ideation was defined as a score of 1 [seldom] or higher on either the suicidal thoughts or the suicidal plans item for the last 12 months period, or as a score of 1 [present] on the suicidal thoughts or suicidal plans items for the last two weeks. Additionally, one item measured the occurrence of suicidal attempts [0 = no/1 = yes].

The life-time occurrence of non-suicidal self-injury (NSSI) [0 = no/1 = yes] was assessed using the respective item of the *Self-Injurious Thoughts and Behavior Interview (SITBI)* [22].

#### Help-seeking

Attitudes towards help-seeking for mental health problems were assessed using the *Inventory of Attitudes Towards Seeking Mental Health Services (IASMHS)* [23]. This questionnaire comprises 24 items scored from 0 [do not agree] to 4 [agree] and can be classified into three subscales, with eight items each: (a) psychological openness, which evaluates a person’s willingness to acknowledge and seek professional help for mental health problems, (b) help-seeking propensity, which measures the extent to which a person believes he or she is capable of seeking professional help and how strongly he or she is personally inclined to do so, and (c) indifference to stigma, which assesses how much a person worries about what other people might think, if they found out, that he or she is seeking professional help.

Actual help-seeking behavior was assessed by using the *Actual Help-Seeking Questionnaire (AHSQ)* [24] containing 13 items. The first question asks whether the individual has ever sought help for a mental health problem [0 = no; 1 = yes, in the last 12 months; 2 = yes, but it has been longer than 12 months]. In the case of help-seeking, items 2–13 list various forms of professional and informal help that can be marked as used [1] or not used [0]. Dichotomous items [0 = no; 1 = yes, in the last 12 months or more than 12 months ago] for professional [i.e., item 2 (partner), 3 (friend), 4 (parent), 5 (other family member)] and informal [i.e., item 6 (school psychologist/social worker), 7 (psychotherapist), 8 (psychiatrist), 9 (counselling centre), 10 (telephone counselling), 11 (family doctor), 12 (teacher)] help were used for the current analysis [24, 25].

Barriers to help-seeking behavior were surveyed using a 14-item questionnaire that was based on an extensive literature review and specifically designed for the purpose of the ProHEAD consortium. The first item asked whether the individual would seek professional help for mental health problems and was dichotomous [0 = no/1 = yes]. If “no” was indicated, items 2 to 14 were presented listing potential reasons for the lack of help-seeking behavior. These items were scored from 0 [not true] to 3 [true]. The mean score, indicating more perceived barriers to seeking help, was included in the current analysis.

### Data analysis

**Fig. 1:**
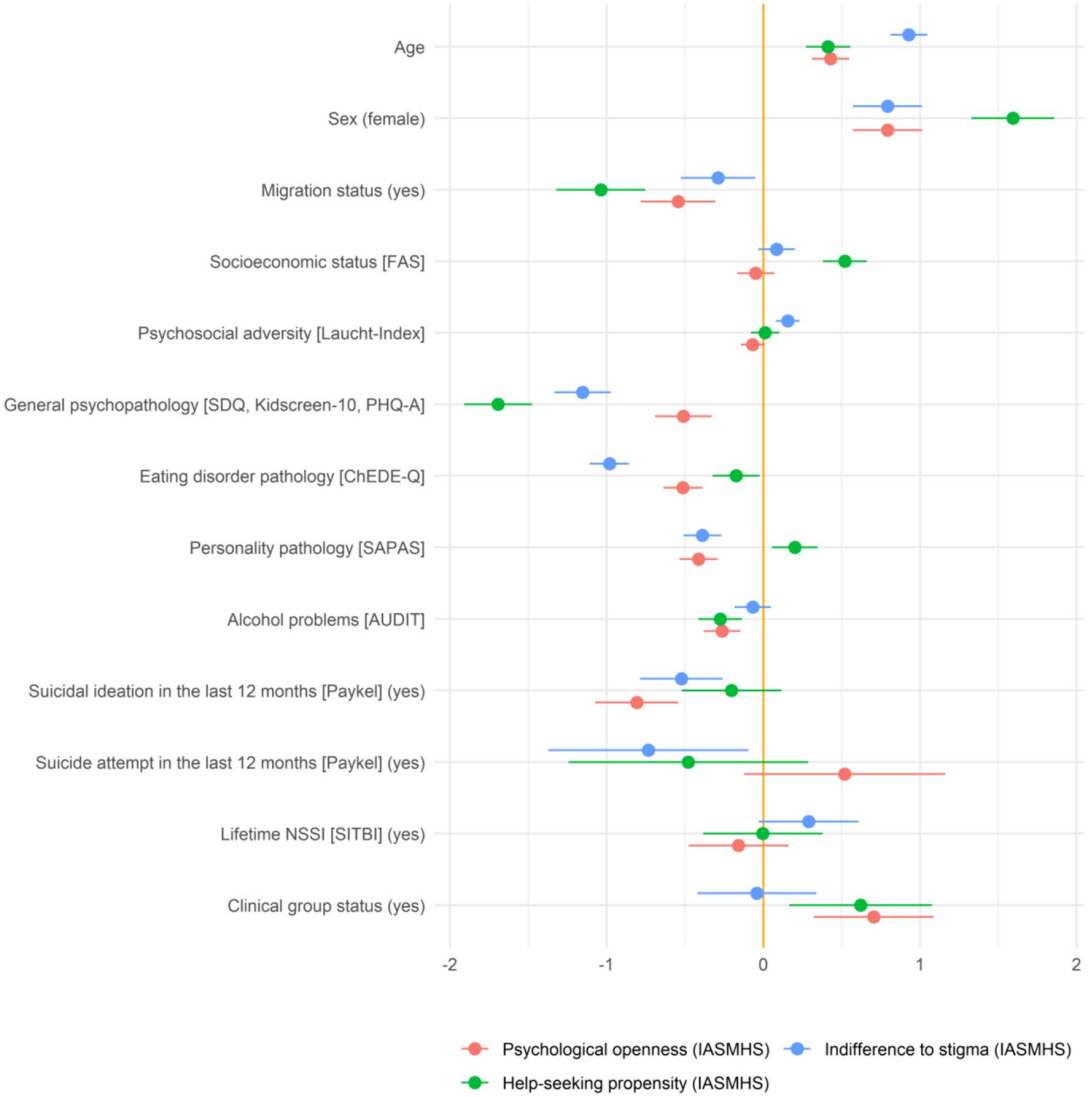
Regression coefficients and 95% confidence intervals for each predictor for help-seeking attitudes (IASMHS subscales). Continuous predictors were standardized (by one standard deviation). The predictor “center” and the subgroup “migration status unknown” (*n* = 25) are not included

Participants were allocated to the clinical group or non-clinical group, respectively, reflecting whether they reached a predefined threshold for relevant mental health problems or not. The threshold reflects the allocation criteria to the first RCT of the ProHEAD consortium (i.e., ProHEAD Online by Kaess et al. [26]), which are detailed in Online Resource 1. Pearson correlations between all variables revealed a high overlap between the SDQ, the KIDSCREEN-10, and the PHQ-A (see Fig. 1 in Online Resource 1). Accordingly, a composite score reflecting “general psychopathology” was created by summing up the z-standardized scores of the three scales. Notably, the KIDSCREEN-10 score was multiplied by − 1 before the composite score was built. For descriptive purposes, the number and percentages for categorical variables and the mean and standard deviations for continuous variables were calculated.

To explore predictors of help-seeking attitudes and behavior, three linear regression analyses were conducted for IASMHS psychological openness, help-seeking propensity, and indifference to stigma, and three logistic regression analyses for AHSQ actual help-seeking, professional help-seeking, and informal help-seeking as outcome variables, respectively. Age, gender, center, migration status, socioeconomic status (FAS), psychosocial adversity (Laucht-Index), general psychopathology (composite score derived from SDQ, KIDSCREEN-10, and PHQ-A), eating disorder psychopathology (ChEDE-Q), personality pathology (SAPAS), alcohol problems (AUDIT), suicidal ideation (Paykel), suicide attempts (Paykel), and NSSI (SITBI) were entered as predictor variables into the regression analyses. In the regression analyses with the AHSQ variables as outcomes, the IASMHS subscales and the barriers to help-seeking questionnaire were considered as additional predictors. Continuous predictor variables were z-standardized to facilitate comparison between predictors. The importance of single predictor variables was determined using the LMG formula [27]. LMG calculates the relative contribution of each predictor to the explained total variance (R^2^) for linear regressions and the explained Mc Fadden’s pseudo R^2^ for logistic regressions, respectively, with the consideration of the sequence of predictors appearing in the model. The explained variance (in % of the total variance) or pseudo R^2^ by each predictor variable reflecting its relative importance estimated by LMG is presented in the results below. The regression coefficients for linear regression and Odds Ratios (OR) for logistic regressions, respectively, representing the direction and strength of the relationships between predictor and outcome variables can be found in Online Resource 1. Finally, to explore whether the importance of predictor variables shows differences between adolescents who reached the threshold for relevant mental health problems and those who did not, all regression analyses were repeated including interaction terms clinical group-status × predictor for all predictor variables. The inclusion of the interaction terms allows the examination of the clinical group-status as a moderator of the associations between the predictor variables and the outcomes. As suggested by a reviewer, the regression analyses predicting help-seeking attitudes (IASMHS subscales) and actual help-seeking behavior (AHSQ subscales) were repeated with the SDQ subscales (i.e., conduct problems, emotional symptoms, hyperactivity/inattention, peer relationship problems, and prosocial behavior), the KIDSCREEN-10 and the PHQ-9 as individual predictors instead of the general psychopathology composite score. Full results of these additional analyses are provided in Online Resource 1 (Tables 3–6). The overall patterns of findings remained unchanged. All analyses were run with R [28] using r packages relaimpo and sensitivity for LMG calculations.

## Results

### Sample characteristics

Out of 767 invited schools, 185 (24.12%) did not respond, 378 declined (49.28%), and 195 (25.42%) agreed to participate in the study. While 45’084 adolescents were invited to take part in the study, 9954 (22.08%) initiated, and 9509 (21.09%) completed the initial screening assessment, and were, thus, included in the current study.

A total of 1606 participants (16.9%) showed clinically relevant mental health problems (i.e., the clinical group). Of those adolescents, 895 (55.7%) reported having sought help (in a lifetime), with higher rates observed for informal (*n* = 834, 51.9%) compared to professional (*n* = 411, 25.6%) sources. Detailed sociodemographic and clinical data of the whole sample as well as of the clinical and non-clinical groups are provided in Table 1.

**Table 1:** Sociodemographic and clinical characteristics of the sample (*N* = 9509)

	Clinical group (*n* = 1606; 16.9%)	Non-clinical group (*n* = 7903; 83.1%)	Overall (*N* = 9509)
Age (years); mean (SD)	15.5 (2.40)	15.0 (2.35)	15.1 (2.37)
Sex (female); n (%)	1212 (75.5)	4363 (55.2)	5575 (58.6)
Center			
Heidelberg, n (%)	465 (29.0)	2167 (27.4)	2632 (27.7)
Hamburg, n (%)	321 (20.0)	1507 (19.1)	1828 (19.2)
Leipzig, n (%)	371 (23.1)	1823 (23.1)	2194 (23.1)
Schwäbisch Gmünd, n (%)	291 (18.1)	1689 (21.4)	1980 (20.8)
Marburg, n (%)	158 (9.8)	717 (9.1)	875 (9.2)
School type			
Gymnasium, n (%)	729 (45.4)	4313 (54.6)	5042 (53.0)
Realschule, n (%)	172 (10.7)	823 (10.4)	995 (10.5)
Haupt- und Werkrealschulen, n (%)	130 (8.1)	459 (5.8)	589 (6.2)
Gemeinschaftsschulen (Oberschule, Stadtteilschule), n (%)	316 (19.7)	1344 (17.0)	1660 (17.5)
Berufsschule, n (%)	204 (12.7)	746 (9.4)	950 (10.0)
Other, n (%)	50 (3.1)	185 (2.3)	235 (2.5)
Missing, n (%)	5 (0.3)	33 (0.4)	38 (0.4)
Migration status			
No, n (%)	965 (60.1)	5442 (68.9)	6407 (67.4)
Yes, n (%)	584 (36.4)	2299 (29.1)	2883 (30.3)
Unknown	57 (3.5)	162 (2.0)	219 (2.3)
Socioeconomic status [FAS]			
Mean (SD)	5.96 (1.89)	6.61 (1.79)	6.50 (1.82)
Low, n (%)	171 (2.2)	68 (4.2)	239 (2.5)
Medium, n (%)	1801 (22.8)	533 (33.2)	2334 (24.5)
High, n (%)	5931 (75.0)	1005 (62.6)	6936 (72.9)
Psychosocial adversity [Laucht-Index]			
Mean (SD)	2.38 (1.84)	1.31 (1.50)	1.49 (1.61)
No risk, n (%)	285 (17.7)	3312 (41.9)	3597 (37.8)
Low risk, n (%)	632 (39.4)	3058 (38.7)	3690 (38.8)
High risk, n (%)	689 (42.9)	1533 (19.4)	2222 (23.4)
General psychopathology [SDQ, Kidscreen-10, PHQ-A]; mean (SD)	4.18 (2.00)	− 0.849 (1.95)	0.00 (2.72)
Eating disorder pathology [ChEDE-Q]; mean (SD)	2.03 (1.54)	0.765 (0.953)	0.979 (1.18)
Personality pathology [SAPAS]; mean (SD)	4.06 (1.42)	2.70 (1.32)	2.93 (1.43)
Alcohol problems [AUDIT]; mean (SD)	4.50 (6.69)	2.09 (3.65)	2.49 (4.41)
Suicidal ideation in the last 12 months [Paykel]; yes (n, %)	1233 (76.8)	2033 (25.7)	3266 (34.3)
Suicidal ideation in the last 2 weeks [Paykel]; yes (n, %)	752 (46.8)	488 (6.2)	1240 (13.0)
Suicide attempt in the last 12 months [Paykel]; yes (n, %)	190 (11.8)	84 (1.1)	274 (2.9)
Suicide attempt in the last 2 weeks [Paykel]; yes (n, %)	50 (3.1)	10 (0.1)	60 (0.6)
Lifetime NSSI [SITBI]; yes (n, %)	806 (50.2)	872 (11.0)	1678 (17.6)
Psychological openness [IASMHS]; mean (SD)	24.7 (5.27)	26.3 (5.17)	26.0 (5.22)
Help-seeking propensity [IASMHS]; mean (SD)	24.1 (6.11)	26.8 (6.29)	26.3 (6.34)
Indifference to stigma [IASMHS]; mean (SD)	28.4 (6.36)	31.8 (5.08)	31.2 (5.47)
Actual help-seeking [AHSQ]; yes (n, %)	895 (55.7)	1948 (24.6)	2843 (29.9)
Searched for professional help [AHSQ]; yes (n, %)	411 (25.6)	707 (8.9)	1118 (11.8)
Searched for informal help [AHSQ]; yes (n, %)	834 (51.9)	1861 (23.5)	2695 (28.3)
Barriers for help-seeking behavior; mean (SD)	8.86 (10.0)	4.15 (7.21)	4.94 (7.95)

School type: After 4 years of elementary school the German school system branches into three types of secondary schools. The so called *Haupt- & Werkrealschulen* (Secondary General School which takes 5 years after Primary School) prepares pupils for vocational training, whereas the *Realschule* (Intermediate Secondary School) concludes with a general certificate of secondary education after 6 years. Eight years of *Oberschule*, * Gymnasium* provide pupils with a general university entrance qualification; *Gemeinschaftsschulen* are secondary schools in Saxony. *Berufsschulen* are schools that provide education to students while undergoing an apprenticeship

*AUDIT* Alcohol Use Disorders Identification Test, *ChEDE-Q* Eating Disorder Examination-Questionnaire for Children, *FAS* Family Affluence Scale, *NSSI* non-suicidal self-injury, *PHQ-A* Patient Health Questionnaire- 9 modified for adolescents, *SAPAS* Self-Rated Standardized Assessment of Personality-Abbreviated Scale, *SDQ* Strengths and Difficulties Questionnaire, *SITBI* Self-Injurious Thoughts and Behavior Interview

#### Correlates of attitudes toward seeking mental health services

All sociodemographic and clinical factors together explained 15.84% of the variance of indifference to stigma, 9.91% of the variance of help-seeking propensity, and 6.84% of the variance of psychological openness. The respective proportions of the variances explained by each predictor reflecting their relative importance are shown in Table 2. The regression coefficients (and their 95% confidence intervals) are presented in Table 1 in Online Resource 1 and illustrated in Fig. 1.

**Table 2:** Respective proportions (%) of the total variance (R^2^) of psychological openness, help-seeking propensity, and indifference to stigma (IASMHS) explained by each predictor

Predictor variables	Psychological openness	Help-seeking propensity	Indifference to stigma
Age	0.31	0.19	2.03
Sex	0.29	1.04	0.25
Center	0.52	0.57	0.12
Migration status	0.23	1.00	0.16
Socioeconomic status [FAS]	0.03	1.02	0.06
Psychosocial adversity [Laucht-Index]	0.23	0.37	0.17
General psychopathology [SDQ, Kidscreen-10, PHQ-A]	1.22	3.26	4.02
Eating disorder pathology [ChEDE-Q]	1.22	0.47	3.92
Personality pathology [SAPAS]	1.01	0.25	1.51
Alcohol problems [AUDIT]	0.20	0.16	0.09
Suicidal ideation in the last 12 months [Paykel]	0.97	0.47	1.26
Suicide attempt in the last 12 months [Paykel]	0.05	0.17	0.38
Lifetime NSSI [SITBI]	0.29	0.26	0.45
Clinical group status	0.29	0.68	1.43
Total explained variance (R^2^)	6.84	9.91	15.84

*AUDIT* Alcohol Use Disorders Identification Test, *ChEDE-Q* Eating Disorder Examination-Questionnaire for Children, *FAS* Family Affluence Scale, *IASMHS* Inventory of Attitudes toward Seeking Mental Health Services, *NSSI* non-suicidal self-injury, *PHQ-A* Patient Health Questionnaire-9 modified for adolescents, *SAPAS* Self-Rated Standardized Assessment of Personality-Abbreviated Scale, *SDQ* Strengths and Difficulties Questionnaire, *SITBI* Self-Injurious Thoughts and Behavior Interview

#### Correlates of actual help-seeking behavior

**Fig. 2:**
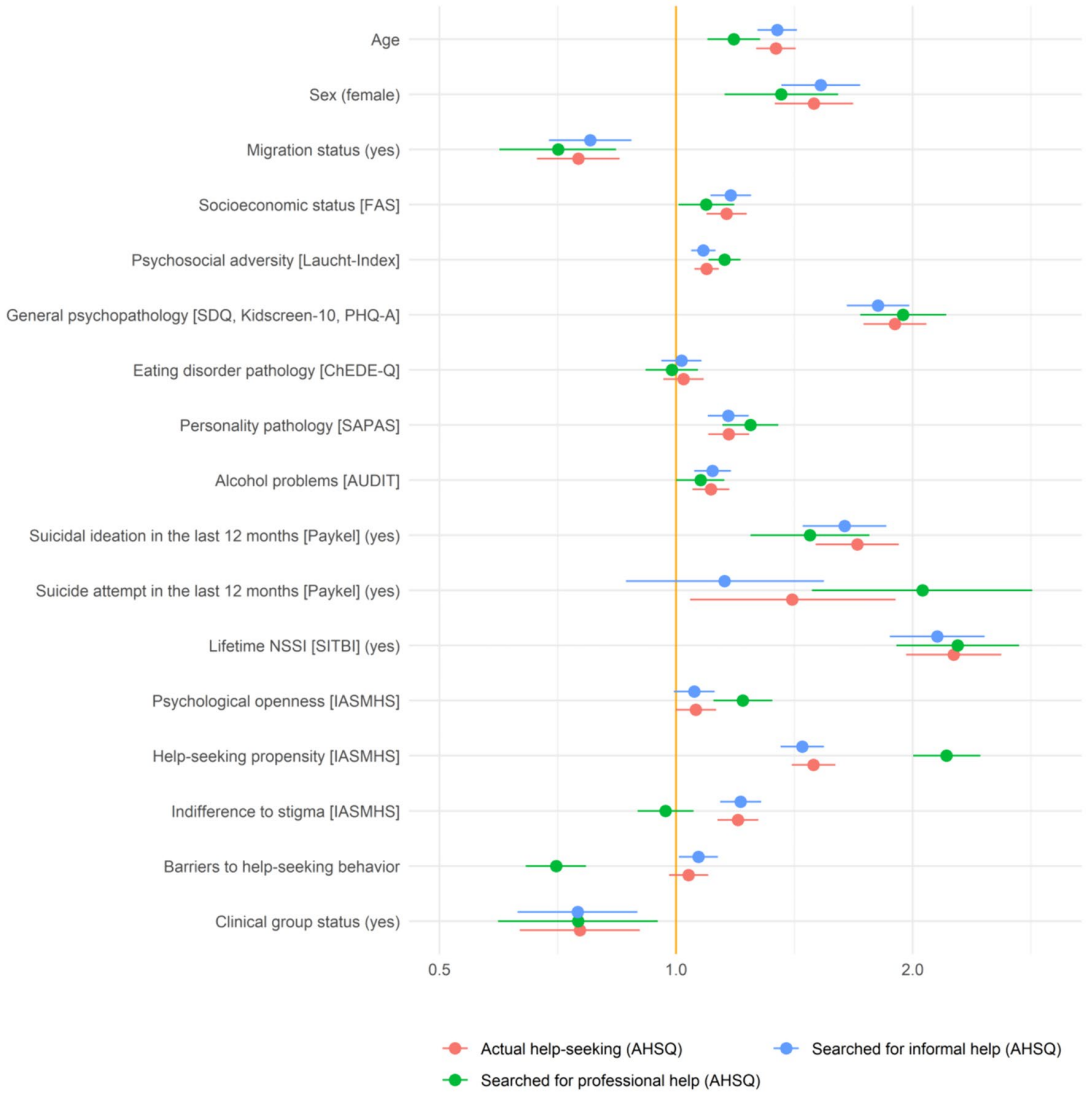
Odds ratios (OR) and 95% confidence intervals for each predictor for help-seeking behavior (AHSQ subscales). Continuous predictors were standardized (by one standard deviation). The predictor “center” and the subgroup “migration status unknown” (*n* = 25) are not included

Sociodemographic, clinical, and attitudinal factors as well as perceived barriers had pseudo R^2^ values of 20.44% for actual help-seeking behavior, 22.62% for professional help-seeking, and 18.75% for informal help-seeking. The respective proportions of the pseudo R^2^ explained by each predictor reflecting their relative importance are given in Table 3. The OR (and their 95% confidence intervals) are presented in Table 2 in Online Resource 1 and illustrated in Fig. 2.

**Table 3:** Respective proportions (%) of the pseudo R^2^ for actual help-seeking, professional help-seeking, and informal help-seeking (AHSQ) explained by each predictor

Predictor variables	Actual help-seeking	Professional help-seeking	Informal help-seeking
Age	1.70	0.78	1.75
Sex	1.14	0.78	1.17
Center	0.30	0.24	0.26
Migration status	0.28	0.27	0.25
Socioeconomic status [FAS]	0.16	0.10	0.17
Psychosocial adversity [Laucht-Index]	0.79	1.13	0.67
General psychopathology [SDQ, Kidscreen-10, PHQ-A]	4.36	3.85	3.84
Eating disorder pathology [ChEDE-Q]	0.93	0.65	0.82
Personality pathology [SAPAS]	1.40	1.54	1.30
Alcohol problems [AUDIT]	0.75	0.36	0.77
Suicidal ideation in the last 12 months [Paykel]	2.45	1.56	2.18
Suicide attempt in the last 12 months [Paykel]	0.28	0.63	0.18
Lifetime NSSI [SITBI]	2.91	2.63	2.65
Psychological openness [IASMHS]	0.06	0.45	0.05
Help-seeking propensity [IASMHS]	1.30	4.77	1.13
Indifference to stigma [IASMHS]	0.39	0.18	0.42
Barriers to help-seeking behavior	0.15	1.63	0.17
Clinical group status	1.11	1.10	0.96
Total explained pseudo R^2^	20.44	22.62	18.75

*AHSQ* Actual Help-Seeking Questionnaire, *AUDIT* Alcohol Use Disorders Identification Test, *ChEDE-Q* Eating Disorder Examination-Questionnaire for Children, *FAS* Family Affluence Scale, *IASMHS* Inventory of Attitudes Towards Seeking Mental Health Services, *PHQ-A* Patient Health Questionnaire-9 modified for adolescents, *SAPAS* Self-Rated Standardized Assessment of Personality-Abbreviated Scale, *SDQ* Strengths and Difficulties Questionnaire, *SITBI* Self-Injurious Thoughts and Behavior Interview

#### Moderation by clinical group-status

The additional amount of variance or pseudo R^2^ for the outcome variables explained by the interaction terms clinical group status × predictor (for all variables) ranged between 0.003 and 0.007 (see Table 4). This indicates that the moderation of the associations between the sociodemographic and clinical variables and attitudes towards or actual help-seeking behavior, respectively, by the clinical group status was negligible.

**Table 4:** Total explained variance or pseudo R^2^, respectively, of attitudes towards seeking mental health services (IAMHS subscales) and actual help-seeking behavior (AHSQ subscales) by (A) all predictors, and (B) when the interactions “clinical group status x predictor” (for all variables) were additionally considered

Outcome variables	(A) Without interactions	(B) With interactions	Likelihood ratio test
Psychological openness [IASMHS]	0.068	0.073	0.0000221
Help-seeking propensity [IASMHS]	0.099	0.105	0.0000005
Indifference to stigma [IASMHS]	0.158	0.161	0.0187750
Actual help-seeking [AHSQ]	0.204	0.210	0.0000007
Professional help-seeking [AHSQ]	0.226	0.232	0.0148296
Informal help-seeking [AHSQ]	0.187	0.194	0.0000003

*AHSQ* Actual Help-Seeking Questionnaire, *IASMHS* Inventory of Attitudes Towards Seeking Mental Health Services

## Discussion

In a large sample of German adolescents aged 12–25, 16.9% showed clinically relevant mental health symptoms according to the results of an online self-report screening. This matches the recently reported prevalence rates of mental disorders among children and adolescents in Europe [2]. Also, 17.6% reported lifetime NSSI, which is consistent with the meta-analytic finding that approximately one in five adolescents engage in deliberate self-harm over time [29]. Moreover, 34.3% of the adolescents reported suicidal ideation and 2.9% a suicide attempt in the previous 12 months. These 12-month prevalence rates are somewhat lower than the reported life-time prevalence rates for suicidal thoughts and attempts in young people [30]. Strikingly, while 55.7% of the young people with clinically relevant mental health symptoms reported having sought help (lifetime), substantially more did so from informal (51.9%) than from professional (25.6%) sources, confirming the previously reported low professional help-seeking behavior of this age group [6, 7].

A positive attitude towards professional help-seeking, reflected in higher help-seeking propensity scores, emerged as the most important factor explaining variability in professional help-seeking behavior, followed by perceived barriers to mental health help-seeking and clinical factors (i.e., increased levels of general psychopathology, NSSI, and suicidal ideations in the last 12 months). This aligns with previous findings that attitudes towards help-seeking and perceived barriers and facilitators significantly influence professional help-seeking [31, 32]. Clinical factors had, overall, a greater impact compared to sociodemographic factors on both, help-seeking attitudes, and actual help-seeking behavior, potentially indicating that the level of psychopathological distress is a key determinant of help-seeking. Therefore, initiatives aimed at increasing help-seeking behavior, especially among young people with high levels of psychopathological distress, should primarily focus on fostering positive attitudes towards professional help-seeking and reducing perceived barriers. This approach is crucial for minimizing the duration of untreated illness, which is associated with poor outcomes [33–35]. Consistent with previous findings [11, 36–38], higher SES, female gender, and older age were related to more positive attitudes towards professional help-seeking, and with an increased likelihood to seek professional help. The influence of SES and age may be attributed to differences in mental health literacy (which tend to increase with SES and age) [39], while the gender effect could be explained by gender-specific norms and socialization patterns [36]. Interestingly, higher levels of psychopathology (i.e., general, eating disorder, and personality pathology, NSSI, and suicidal ideation) were related to more negative attitudes (as reflected in lower levels of psychological openness, help-seeking propensity, and indifference to stigma), but increased likelihood for professional help-seeking behavior. These findings raise the question whether previous negative experiences with mental health services have contributed to the negative perception about professional help, as previously reported [40–42]. For instance, experiencing prior treatment as stigmatizing or unhelpful may exacerbate feelings of hopelessness and distrust toward mental health professionals. Finally, the results of the moderation analyses indicate that the relevance of sociodemographic and clinical predictors for both attitudes towards help-seeking and actual help-seeking behavior were comparable for adolescents with clinically relevant mental problems (i.e., the clinical group) and those without (i.e., the non-clinical group). However, the interpretation of this finding remains complex, as the clinical group-status overlaps with clinical variables (i.e., general and eating disorder psychopathology, and alcohol problems) that were identified as important predictors of attitudes towards help-seeking and actual help-seeking behavior.

Several limitations must be considered: First, the representativeness of the sample is limited, because recruitment took place in five pre-selected regions in Germany and was dependent on the agreement of schools and students to participate. The Covid-19 pandemic as well as the low participation rate at student level made it necessary to invite more schools than originally planned (e.g., also vocational schools). A recent analysis conducted at one of the study centers identified that a primary reason for non-participation was a lack of concern about the topic of “mental health” [43]. This suggests that students who chose not to participate might have experienced fewer mental health problems compared to those who did participate in the study. Alternatively, these findings may reflect a fear of stigma related to mental health, which presents a major barrier to help-seeking. The representativeness of the sample is further constrained by including only male and female genders, which limits the generalizability of the study results to non-binary adolescents. Future research should explore gender diversity as a predictor of help-seeking attitudes and behaviors. Second, the clinical group-status of participants was based on self-report, which is not equivalent to a psychiatric diagnosis as obtained through a structured clinical interview with an adolescent and their parents. In addition, the determination of the clinical group-status in this study is somewhat arbitrary. The chosen criteria align with the allocation criteria to the first RCT of the ProHEAD consortium [26]. Notably, the initial allocation criteria were adjusted after a preliminary analysis of 10% of the sample data revealed that they were too inclusive. The adequacy of these adjusted criteria is supported by the fact that the frequencies of mental health problems in the current study correspond well with prevalence rates reported previously [2]. Third, recent social and economic changes may have affected the FAS’ ability to accurately measure adolescent SES. These changes include climate change, which may alter travel patterns (potentially influencing the car ownership item), the COVID-19 pandemic, which led to travel restrictions and a shift to online education and remote work (potentially influencing the holiday and computer items), or technological advances making personal computers more affordable and therefore less suitable as an indicator for adolescent SES [44, 45] Future research should consider multiple SES indicators when predicting help-seeking attitudes and behaviors. Fourth, the predictor variables considered in the current analyses explained less variance in help-seeking attitudes (IASMHS subscales) compared to help-seeking behavior (AHSQ subscales). This suggests that other variables not assessed in the current study, such as peer influence or exposure to mental health education, may play a significant role in shaping attitudes towards seeking professional mental health help. These factors could be important areas for future research. Finally, as the current study was cross-sectional, correlates of mental health help-seeking attitudes and behavior were examined, which does not allow for causal conclusions.

To conclude, the findings of the current study confirm attitudinal aspects, perceived barriers, as well as clinical and sociodemographic characteristics as relevant factors for the understanding of why some young people with mental health problems do seek professional help, while others do not [9–11]. They extend previous research by demonstrating that the individual propensity and capacity to seek professional help is most relevant, followed by severity of psychopathology and perceived barriers (e.g., lack of knowledge of mental health services or lack of time resources, travel distances, and confidentiality concerns), while sociodemographic factors such as SES, age, and gender are of minor relevance. These findings overall carry noteworthy socio-political implications, as they emphasize that action is needed to enable and empower young people to seek professional help (e.g., through classroom-based interventions to increase mental health literacy), and to reduce stigma (e.g., through mental health campaigns) and structural barriers to mental health treatment [9, 26].

## Electronic supplementary material

Below is the link to the electronic supplementary material.

Supplementary Material 1

## Data Availability

Data is available upon on request from the corresponding author.

## References

[1] Solmi M, Radua J, Olivola M et al (2022) Age at onset of mental disorders worldwide: large-scale meta-analysis of 192 epidemiological studies. Mol Psychiatry 27:281–295. 10.1038/s41380-021-01161-7 34079068 10.1038/s41380-021-01161-7 PMC8960395

[2] Sacco R, Camilleri N, Eberhardt J et al (2022) A systematic review and meta-analysis on the prevalence of mental disorders among children and adolescents in Europe. Eur Child Adolesc Psychiatry. 10.1007/s00787-022-02131-2 36581685 10.1007/s00787-022-02131-2 PMC9800241

[3] Mulraney M, Coghill D, Bishop C et al (2021) A systematic review of the persistence of childhood mental health problems into adulthood. Neurosci Biobehav Rev 129:182–205. 10.1016/j.neubiorev.2021.07.030 34363845 10.1016/j.neubiorev.2021.07.030

[4] Castelpietra G, Knudsen AKS, Agardh EE et al (2022) The burden of mental disorders, substance use disorders and self-harm among young people in Europe, 1990–2019: findings from the Global Burden of Disease Study 2019. Lancet Reg Health Eur 16:100341. 10.1016/j.lanepe.2022.100341 35392452 10.1016/j.lanepe.2022.100341 PMC8980870

[5] Rickwood T (2012) Conceptual measurement framework for help-seeking for mental health problems. Psychol Res Behav Manag 5:173–183. 10.2147/PRBM.S38707 23248576 10.2147/PRBM.S38707 PMC3520462

[6] Lustig S, Kaess M, Schnyder N, Michel C, Brunner R, Tubiana A, Kahn JP, Sarchiapone M, Hoven CW, Barzilay S, Apter A, Balazs J, Bobes J, Saiz PA, Cozman D, Cotter P, Kereszteny A, Podlogar T, Postuvan V, Värnik A, Resch F, Carli V, Wasserman D (2023) The impact of school-based screening on service use in adolescents at risk for mental health problems and risk-behaviour. Eur Child Adolesc Psychiatry 32(9):1745–1754. 10.1007/s00787-022-01990-z 35488938 10.1007/s00787-022-01990-z PMC10460322

[7] Kaess M, Schnyder N, Michel C et al (2022) Twelve-month service use, suicidality and mental health problems of European adolescents after a school-based screening for current suicidality. Eur Child Adolesc Psychiatry 31:229–238. 10.1007/s00787-020-01681-7 33320300 10.1007/s00787-020-01681-7 PMC8837507

[8] Yonemoto N, Kawashima Y (2023) Help-seeking behaviors for mental health problems during the COVID-19 pandemic: a systematic review. J Affect Disord 323:85–100. 10.1016/j.jad.2022.11.043 36435398 10.1016/j.jad.2022.11.043 PMC9684094

[9] Aguirre Velasco A, Cruz ISS, Billings J et al (2020) What are the barriers, facilitators and interventions targeting help-seeking behaviours for common mental health problems in adolescents? A systematic review. BMC Psychiatry 20:293. 10.1186/s12888-020-02659-0 32527236 10.1186/s12888-020-02659-0 PMC7291482

[10] Radez J, Reardon T, Creswell C et al (2021) Why do children and adolescents (not) seek and access professional help for their mental health problems? A systematic review of quantitative and qualitative studies. Eur Child Adolesc Psychiatry 30:183–211. 10.1007/s00787-019-01469-4 31965309 10.1007/s00787-019-01469-4 PMC7932953

[11] Reardon T, Harvey K, Baranowska M et al (2017) What do parents perceive are the barriers and facilitators to accessing psychological treatment for mental health problems in children and adolescents? A systematic review of qualitative and quantitative studies. Eur Child Adolesc Psychiatry 26:623–647. 10.1007/s00787-016-0930-6 28054223 10.1007/s00787-016-0930-6 PMC5446558

[12] Kaess M, Bauer S (2019) Editorial promoting help-seeking using E-Technology for ADolescents: the ProHEAD consortium. Trials 20:72. 10.1186/s13063-018-3162-x 30670060 10.1186/s13063-018-3162-x PMC6341557

[13] Boyce W, Torsheim T, Currie C, Zambon A (2006) The Family Affluence Scale as a measure of National Wealth: validation of an adolescent self-report measure. Soc Indic Res 78:473–487. 10.1007/s11205-005-1607-6

[14] Laucht M, Esser G, Schmidt MH et al (1992) Risikokinder: Zur Bedeutung biologischer und psychosozialer Risiken für die kindliche Entwicklung in den beiden ersten Lebensjahren. Praxis Der Kinderpsychologie Und Kinderpsychiatrie 41:275–285 1279655

[15] Ravens-Sieberer U, Gosch A, Rajmil L et al (2005) KIDSCREEN-52 quality-of-life measure for children and adolescents. Expert Rev Pharmacoecon Outcomes Res 5:353–364. 10.1586/14737167.5.3.353 19807604 10.1586/14737167.5.3.353

[16] Goodman A, Goodman R (2009) Strengths and Difficulties Questionnaire as a dimensional measure of child mental health. J Am Acad Child Adolesc Psychiatry 48:400–403. 10.1097/CHI.0b013e3181985068 19242383 10.1097/CHI.0b013e3181985068

[17] Johnson JG, Harris ES, Spitzer RL, Williams JBW (2002) The Patient Health Questionnaire for adolescents. J Adolesc Health 30:196–204. 10.1016/S1054-139X(01)00333-0 11869927 10.1016/s1054-139x(01)00333-0

[18] Hilbert A, Tuschen-Caffier B (2016) Eating Disorder Examination-Questionnaire: Deutschsprachige Übersetzung. dgvt, Tübingen

[19] Hesse M, Moran P (2010) Screening for personality disorder with the Standardised Assessment of Personality: Abbreviated Scale (SAPAS): further evidence of concurrent validity. BMC Psychiatry 10:10. 10.1186/1471-244X-10-10 20109169 10.1186/1471-244X-10-10 PMC2824652

[20] Saunders JB, Aasland OG, Babor TF, de la Fuente JR, Grant M (1993) Development of the Alcohol Use DisordersIdentification Test (AUDIT): WHO collaborative project on early detection of persons with harmful alcoholconsumption–II. Addiction 88(6):791–804. 10.1111/j.1360-0443.1993.tb02093.x (**PMID: 8329970**) 8329970 10.1111/j.1360-0443.1993.tb02093.x

[21] Paykel ES, Myers JK, Lindenthal JJ, Tanner J (1974) Suicidal feelings in the general population: a prevalence study. Br J Psychiatry 124:460–469. 10.1192/bjp.124.5.460 4836376 10.1192/bjp.124.5.460

[22] Fischer G, Ameis N, Parzer P et al (2014) The German version of the Self-Injurious Thoughts and Behaviors Interview (SITBI-G): a tool to assess non-suicidal self-injury and suicidal behavior disorder. BMC Psychiatry 14:265. 10.1186/s12888-014-0265-0 25230931 10.1186/s12888-014-0265-0 PMC4174267

[23] Mackenzie CS, Gekoski WL, Knox VJ (2006) Age, gender, and the underutilization of mental health services: the influence of help-seeking attitudes. Aging Ment Health 10:574–582. 10.1080/13607860600641200 17050086 10.1080/13607860600641200

[24] Rickwood DJ, Braithwaite VA (1994) Social-psychological factors affecting seeking help for emotional problems. Soc Sci Med 39:563–572 7973856 10.1016/0277-9536(94)90099-x

[25] Rickwood D, Deane FP, Wilson CJ, Ciarrochi J (2005) Young people’s help-seeking for mental health problems. Aust E-J Adv Ment Health 4:218–251. 10.5172/jamh.4.3.218

[26] Kaess M, Ritter S, Lustig S et al (2019) Promoting help-seeking using E-technology for ADolescents with mental health problems: study protocol for a randomized controlled trial within the ProHEAD Consortium. Trials 20:94. 10.1186/s13063-018-3157-7 30704534 10.1186/s13063-018-3157-7 PMC6357507

[27] Grömping U (2006) Relative importance for linear regression in R: the Package relaimpo. J Stat Softw. 10.18637/jss.v017.i01

[28] R Core Team (2021) R: a language and environment for statistical computing. R Foundation for Statistical Computing, Vienna

[29] Xiao Q, Song X, Huang L et al (2022) Global prevalence and characteristics of non-suicidal self-injury between 2010 and 2021 among a non-clinical sample of adolescents: a meta-analysis. Front Psychiatry 13:912441. 10.3389/fpsyt.2022.912441 36032224 10.3389/fpsyt.2022.912441 PMC9399519

[30] Van Meter AR, Knowles EA, Mintz EH (2023) Systematic review and meta-analysis: international prevalence of suicidal ideation and attempt in youth. J Am Acad Child Adolesc Psychiatry 62:973–986. 10.1016/j.jaac.2022.07.867 36563876 10.1016/j.jaac.2022.07.867 PMC12702477

[31] Al Omari O, Khalaf A, Al Sabei S et al (2022) Facilitators and barriers of mental health help-seeking behaviours among adolescents in Oman: a cross-sectional study. Nord J Psychiatry 76:591–601. 10.1080/08039488.2022.2038666 35209780 10.1080/08039488.2022.2038666

[32] Ma SON, McCallum SM, Pasalich D et al (2023) Understanding parental knowledge, attitudes and self-efficacy in professional help-seeking for child anxiety. J Affect Disord 337:112–119. 10.1016/j.jad.2023.05.079 37245548 10.1016/j.jad.2023.05.079

[33] Ghio L, Gotelli S, Marcenaro M et al (2014) Duration of untreated illness and outcomes in unipolar depression: a systematic review and meta-analysis. J Affect Disord 152–154:45–51. 10.1016/j.jad.2013.10.002 24183486 10.1016/j.jad.2013.10.002

[34] Penttilä M, Jääskeläinen E, Hirvonen N et al (2014) Duration of untreated psychosis as predictor of long-term outcome in schizophrenia: systematic review and meta-analysis. Br J Psychiatry 205:88–94. 10.1192/bjp.bp.113.127753 25252316 10.1192/bjp.bp.113.127753

[35] Kisely S, Scott A, Denney J, Simon G (2006) Duration of untreated symptoms in common mental disorders: association with outcomes: international study. Br J Psychiatry 189:79–80. 10.1192/bjp.bp.105.019869 16816310 10.1192/bjp.bp.105.019869

[36] Haavik L, Joa I, Hatloy K et al (2019) Help seeking for mental health problems in an adolescent population: the effect of gender. J Ment Health Abingdon Engl 28:467–474. 10.1080/09638237.2017.1340630 10.1080/09638237.2017.1340630 28719230

[37] Horwitz AG, McGuire T, Busby DR et al (2020) Sociodemographic differences in barriers to mental health care among college students at elevated suicide risk. J Affect Disord 271:123–130. 10.1016/j.jad.2020.03.115 32479307 10.1016/j.jad.2020.03.115 PMC7266827

[38] Schomerus G, Appel K, Meffert PJ et al (2013) Personality-related factors as predictors of help-seeking for depression: a population-based study applying the behavioral model of health services use. Soc Psychiatry Psychiatr Epidemiol 48:1809–1817. 10.1007/s00127-012-0643-1 23266663 10.1007/s00127-012-0643-1

[39] González-Sanguino C, Rodríguez-Medina J, Redondo-Pacheco J et al (2024) An exploratory cross-sectional study on mental health literacy of Spanish adolescents. BMC Public Health 24:1469. 10.1186/s12889-024-18933-9 38822283 10.1186/s12889-024-18933-9 PMC11143666

[40] Chandra A, Minkovitz CS (2007) Factors that influence mental health stigma among 8th grade adolescents. J Youth Adolesc 36:763–774. 10.1007/s10964-006-9091-0

[41] Gould MS, Marrocco FA, Hoagwood K et al (2009) Service use by at-risk youths after school-based suicide screening. J Am Acad Child Adolesc Psychiatry 48:1193–1201. 10.1097/CHI.0b013e3181bef6d5 19858758 10.1097/CHI.0b013e3181bef6d5 PMC2891889

[42] Sylwestrzak A, Overholt CE, Ristau KI, Coker KL (2015) Self-reported barriers to treatment engagement: adolescent perspectives from the National Comorbidity Survey-Adolescent supplement (NCS-A). Community Ment Health J 51:775–781. 10.1007/s10597-014-9776-x 25326732 10.1007/s10597-014-9776-x

[43] Baldofski S, Klemm S-L, Kohls E et al (2024) Reasons for non-participation of children and adolescents in a large-scale school-based mental health project. Front Public Health 11:1294862. 10.3389/fpubh.2023.1294862 38259782 10.3389/fpubh.2023.1294862 PMC10800647

[44] Currie C, Alemán Díaz AY, Bosáková L, De Looze M (2024) The International Family Affluence Scale (FAS): charting 25 years of indicator development, evidence produced, and policy impact on adolescent health inequalities. SSM Popul Health 25:101599. 10.1016/j.ssmph.2023.101599 38313871 10.1016/j.ssmph.2023.101599 PMC10835442

[45] Boer M, Moreno-Maldonado C, Dierckens M et al (2024) The implications of the COVID-19 pandemic for the construction of the Family Affluence Scale: findings from 16 countries. Child Indic Res 17:395–418. 10.1007/s12187-023-10082-6

