# Supplementary material for: An examination of sociodemographic and clinical factors influencing help-seeking attitudes and behaviors among adolescents with mental health problems
Source: Eur Child Adolesc Psychiatry. 2024 Aug 27;34(4):1391–402. doi: 10.1007/s00787-024-02568-7 (PMC12000217; doi:10.1007/s00787-024-02568-7)
Supplement: Supplementary file 1 — Supplementary Material 1 [file 787_2024_2568_MOESM1_ESM.docx]

**Online Resource 1 for:**

**An examination of sociodemographic and clinical factors influencing help-seeking attitudes and behaviors among adolescents with mental health problems**

Marialuisa Cavelti^1^, Noemi Anne Ruppen^1^, Silvano Sele^1^, Markus Moessner^3^, Stephanie Bauer^3^, Katja Becker^5^, Jennifer Krämer^5^, Heike Eschenbeck^8^, Christine Rummel-Kluge^4^, Rainer Thomasius^7^, Silke Diestelkamp^7^, Vera Gillé^8^, Sabrina Baldofski^4^, Julian Koenig^6^, the ProHEAD-Consortium, Michael Kaess^1/2^

^1^ University Hospital of Child and Adolescent Psychiatry and Psychotherapy, University of Bern, Bern, Switzerland

^2^ Department of Child and Adolescent Psychiatry, Centre for Psychosocial Medicine, University of Heidelberg, Heidelberg, Germany

^3^ Centre for Psychotherapy Research, Centre for Psychosocial Medicine, University of Heidelberg, Heidelberg, Germany

^4^ Department of Psychiatry and Psychotherapy, Medical Faculty, Leipzig University, Leipzig, Germany

^5^ Department for Child and Adolescent Psychiatry, Psychosomatics and Psychotherapy, Faculty of Medicine, University of Marburg, Marburg, Germany

^6^ University of Cologne, Faculty of Medicine and University Hospital Cologne, Department of Child and Adolescent Psychiatry, Psychosomatics and Psychotherapy, Cologne, German

^7^ German Center for Addiction Research in Childhood and Adolescence, University Hospital Hamburg-Eppendorf, Hamburg, Germany

^8^ Department of Educational Psychology and Health Psychology, University of Education Schwäbisch Gmünd, Schwäbisch Gmünd, Germany

Corresponding author

Michael Kaess, University Hospital for Child and Adolescent Psychiatry and Psychotherapy, Bolligenstrasse 111, 3000 Bern 60, Switzerland, [michael.kaess@upd.ch](mailto:michael.kaess@upd.ch), +41 31 932 85 52

**This online resource includes the following information:**

- Criteria for group allocation (clinical vs. non-clinical group)
- Figure 1: Correlation matrix between sociodemographic and clinical variables
- Table 1: Regression parameters [95% Confidence Interval] for the predictors of psychological openness, help-seeking propensity, and indifference to stigma (IASMHS subscales)
- Table 2: Odds ratios [95% Confidence Interval] for the predictor of actual help-seeking, professional help-seeking and informal help-seeking (AHSQ subscales).
- Additional analyses using the SDQ subscales as predictors, including:
  - Table 3: Respective proportion (n %) of the total variance (R2) of psychological openness, help-seeking propensity, and indifference to stigma (IASMHS) explained by each predictor including the SDQ subscales
  - Table 4: Regression parameters [95% Confidence Interval] for the predictors of psychological openness, help-seeking propensity, and indifference to stigma (IASMHS subscales), including the SDQ subscales
  - Table 5: Respective proportion (n %) of the total variance (R2) of actual help-seeking, professional help-seeking and informal help-seeking (AHSQ subscales) explained by each predictor, including the SDQ subscales
  - Table 6: Regression parameters [95% Confidence Interval] for the predictors of actual help-seeking, professional help-seeking and informal help-seeking (AHSQ subscales) explained by each predictor, including the SDQ subscales
  - Table 7: Respective proportions (in %) of the total variance (R2) of actual help-seeking, professional help-seeking, and informal help-seeking (AHSQ) explained by each predictor.

**Group allocation: clinical group vs. non-clinical group**

Participants were allocated to the clinical group or non-clinical group, respectively, reflecting whether they reached a predefined threshold for relevant mental health problems or not. The threshold reflects the allocation criteria to the first RCT of the ProHEAD consortium (i.e., ProHEAD Online by Kaess et al. [1]): A body mass index (BMI) < 5th percentile (adjusted for age and gender) AND concurrent fear of weight gain (a score of ≥ 3 on item 2 of the *Short Evaluation of Eating Disorder (SEED)* OR a score = 3 on item 9 (reflecting thoughts on self-harm) of the *Patient Health Questionnaire-9 modified for adolescents (PHQ-A)* OR a score of ≥ 20 on the *Alcohol Use Disorders Identification Test (AUDIT)* OR a PHQ-A score above 14 OR a score of ≥ 20 on the *Strengths and Difficulties Questionnaire (SDQ)*.

The SEED [2] contains six items, assessing the key eating disorder symptoms. The second item assessing fear of weight gain was considered for the clinical group-definition. It is scored from 0 [never/not at all] to 4 [always/several times a day].

The PHQ-A [3] measures severity of depressive symptoms within the past two weeks. This questionnaire consists of ten items scored from 0 [not at all/no] to 3 [almost daily/extremely] as well as three items scored from 0 [no] to 1 [yes]. A greater total score means more severe symptoms of depression.

The AUDIT [4] is composed of ten items and assesses signs of alcohol use disorders in the previous 12 months. Its items are coded from 0 [never/1-2 glasses/no] to 4 [four times a week or more/ten or more/yes in the last year]. A greater total score indicates more severe alcohol misuse.

The *SDQ* [5] measures mental health problems in the previous six months. It is a 25-item measure, with each item being scored from 0 [not applicable] to 2 [clearly applicable]. A greater total score indicates more psychological problems.

*Figure 1.* Correlation matrix between sociodemographic and clinical variables.


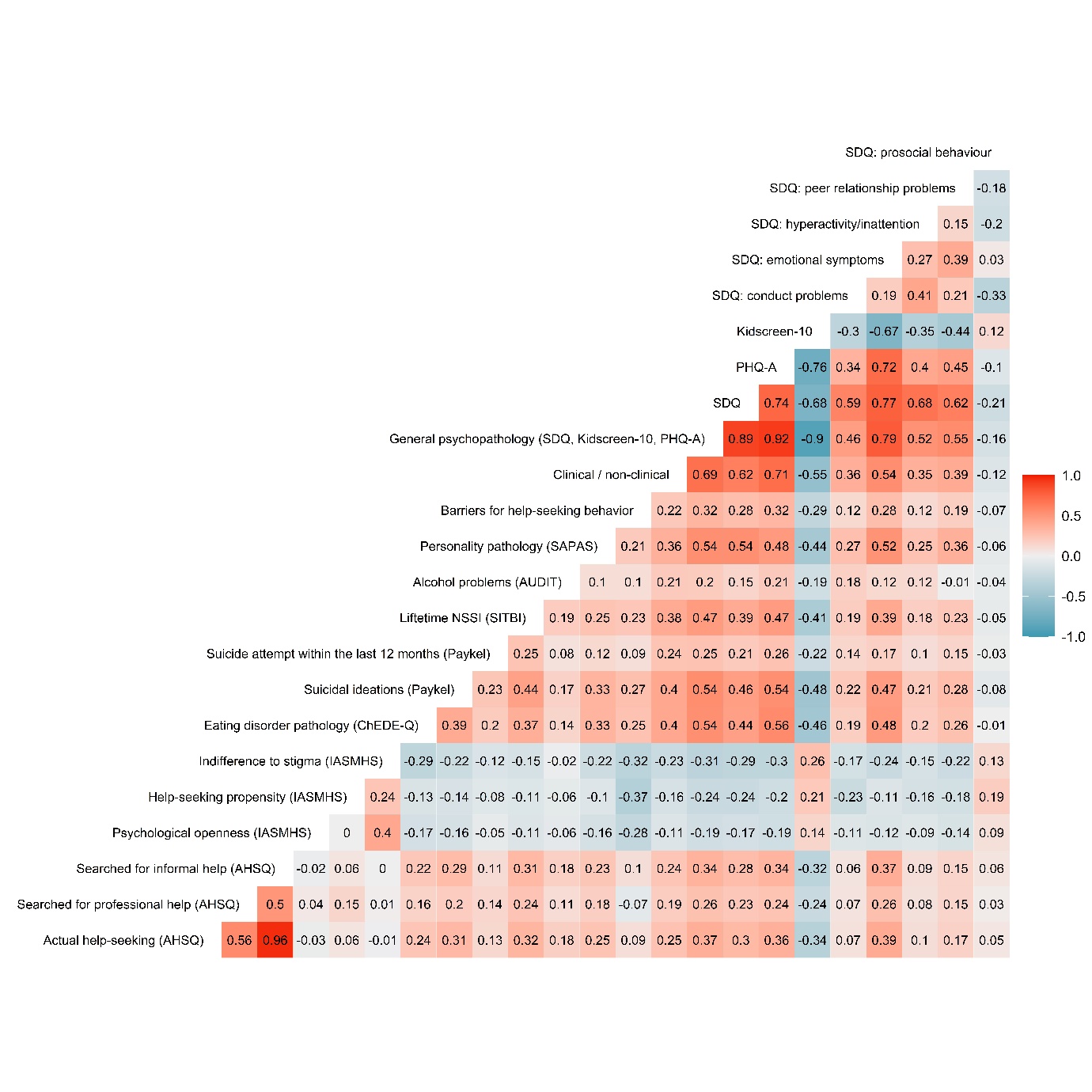


*Notes. AHSQ* Actual Help-Seeking Questionnaire, *AUDIT* Alcohol Use Disorders Identification Test, *ChEDE-Q* Eating Disorder Examination-Questionnaire for children, *IASMHS* Inventory of Attitudes Towards Seeking Mental Health Services, *PHQ-A* Patient Health Questionnaire-9 modified for adolescents, *SAPAS* Self-Rated Standardized Assessment of Personality - Abbreviated Scale, *SDQ* Strengths and Difficulties Questionnaire, *SITBI* Self-Injurious Thoughts and Behavior Interview.

*Table 1.* Regression parameters [95% Confidence Interval] for the predictors of psychological openness, help-seeking propensity, and indifference to stigma (IASMHS subscales).

| Predictor variables | Psychological  openness (IASMHS) | Help-seeking  propensity (IASMHS) | Indifference to stigma (IASMHS) |
| --- | --- | --- | --- |
| Intercept | 26.45 [26.16, 26.74] | 25.30 [24.95, 25.65] | 30.79 [30.50, 31.08] |
| Age | 0.43 [0.31, 0.55] | 0.41 [0.27, 0.55] | 0.93 [0.81, 1.05] |
| Sex [female] | 0.79 [0.57, 1.02] | 1.59 [1.33, 1.86] | 0.79 [0.57, 1.01] |
| Center [Hamburg] | -0.43 [-0.74, -0.12] | 0.67 [0.30, 1.04] | -0.02 [-0.33, 0.29] |
| Center [Leipzig] | -1.09 [-1.38, -0.79] | 0.47 [0.12, 0.82] | 0.19 [-0.10, 0.48] |
| Center [Schwäbisch Gmünd] | -0.53 [-0.82, -0.23] | 0.06 [-0.30, 0.41] | -0.12 [-0.42, 0.17] |
| Center [Marburg] | -0.32 [-0.71, 0.07] | 1.52 [1.06, 1.99] | -0.14 [-0.53, 0.25] |
| Migration background [yes] | -0.54 [-0.78, -0.31] | -1.04 [-1.32, -0.75] | -0.29 [-0.53, -0.05] |
| Migration status unknown [yes] | 0.32 [-0.37, 1.00] | -1.54 [-2.36, -0.72] | -0.28 [-0.96, 0.40] |
| Socioeconomic status [FAS] | -0.05 [-0.17, 0.07] | 0.52 [0.38, 0.66] | 0.08 [-0.03, 0.20] |
| Psychosocial adversity [Laucht-Index] | -0.07 [-0.14, 0.01] | 0.01 [-0.08, 0.10] | 0.16 [0.08, 0.23] |
| General psychopathology [SDQ, Kidscreen-10, PHQ-A] | -0.51 [-0.69, -0.33] | -1.69 [-1.91, -1.48] | -1.15 [-1.33, -0.97] |
| Eating disorder pathology [ChEDE-Q] | -0.51 [-0.64, -0.39] | -0.17 [-0.32, -0.02] | -0.98 [-1.11, -0.86] |
| Personality pathology [SAPAS] | -0.41 [-0.53, -0.29] | 0.20 [0.06, 0.35] | -0.39 [-0.51, -0.27] |
| Alcohol problems [AUDIT] | -0.26 [-0.38, -0.15] | -0.28 [-0.41, -0.14] | -0.07 [-0.18, 0.05] |
| Suicidal ideation in the last 12 months [Paykel] | -0.81 [-1.07, -0.54] | -0.20 [-0.52, 0.11] | -0.52 [-0.79, -0.26] |
| Suicide attempt in the last 12 months [Paykel] | 0.52 [-0.12, 1.16] | -0.48 [-1.24, 0.29] | -0.73 [-1.37, -0.10] |
| Lifetime NSSI [SITBI] | -0.16 [-0.48, 0.16] | -0.00 [-0.39, 0.38] | 0.29 [-0.03, 0.61] |
| Clinical group-status [yes] | 0.71 [0.32, 1.09] | 0.62 [0.16, 1.08] | -0.04 [-0.42, 0.34] |
| Total explained variance (R^2^) | 0.07 | 0.10 | 0.16 |

*Notes: AUDIT* Alcohol Use Disorders Identification Test*, ChEDE-Q* Eating Disorder Examination-Questionnaire for children, *FAS* Family Affluence Scale, *IASMHS* Inventory of Attitudes Towards Seeking Mental Health Services, *NSSI* non-suicidal self-injury, *PHQ-A* Patient Health Questionnaire-9 modified for adolescents, *SAPAS* Self-Rated Standardized Assessment of Personality - Abbreviated Scale, *SDQ* Strengths and Difficulties Questionnaire, *SITBI* Self-Injurious Thoughts and Behavior Interview

*Table 2.* Odds ratios [95% Confidence Interval] for the predictors of actual help-seeking, professional help-seeking and informal help-seeking (AHSQ subscales).

| Predictor variables | Actual help-  seeking (AHSQ) | Professional help-seeking (AHSQ) | Informal help-seeking (AHSQ) |
| --- | --- | --- | --- |
| Intercept | 0.18 [0.16, 0.21] | 0.04 [0.03, 0.05] | 0.17 [0.15, 0.20] |
| Age | 1.34 [1.26, 1.42] | 1.18 [1.10, 1.28] | 1.34 [1.27, 1.42] |
| Sex [female] | 1.50 [1.34, 1.68] | 1.36 [1.15, 1.61] | 1.53 [1.36, 1.71] |
| Center [Hamburg] | 1.21 [1.03, 1.40] | 1.04 [0.84, 1.28] | 1.19 [1.02, 1.38] |
| Center [Leipzig] | 1.15 [1.00, 1.33] | 0.96 [0.78, 1.17] | 1.13 [0.97, 1.30] |
| Center [Schwäbisch Gmünd] | 0.96 [0.82, 1.12] | 0.85 [0.68, 1.05] | 0.96 [0.83, 1.12] |
| Center [Marburg] | 1.05 [0.87, 1.27] | 1.07 [0.84, 1.38] | 1.03 [0.86, 1.25] |
| Migration background [yes] | 0.75 [0.67, 0.85] | 0.71 [0.60, 0.84] | 0.78 [0.69, 0.88] |
| Migration status unknown [yes] | 0.69 [0.49, 0.99] | 0.95 [0.60, 1.52] | 0.68 [0.48, 0.98] |
| Socioeconomic status [FAS] | 1.16 [1.09, 1.23] | 1.09 [1.01, 1.19] | 1.17 [1.11, 1.25] |
| Psychosocial adversity [Laucht-Index] | 1.09 [1.06, 1.13] | 1.15 [1.10, 1.21] | 1.08 [1.05, 1.12] |
| General psychopathology [SDQ, Kidscreen-10, PHQ-A] | 1.90 [1.73, 2.08] | 1.94 [1.71, 2.21] | 1.81 [1.65, 1.98] |
| Eating disorder pathology [ChEDE-Q] | 1.02 [0.96, 1.08] | 0.99 [0.91, 1.07] | 1.02 [0.96, 1.08] |
| Personality pathology [SAPAS] | 1.17 [1.10, 1.24] | 1.24 [1.15, 1.35] | 1.17 [1.10, 1.24] |
| Alcohol problems [AUDIT] | 1.11 [1.05, 1.17] | 1.07 [1.00, 1.15] | 1.11 [1.05, 1.17] |
| Suicidal ideation in the last 12 months [Paykel] | 1.70 [1.51, 1.92] | 1.48 [1.24, 1.76] | 1.64 [1.45, 1.85] |
| Suicide attempt in the last 12 months [Paykel] | 1.40 [1.04, 1.90] | 2.06 [1.49, 2.84] | 1.15 [0.86, 1.54] |
| Lifetime NSSI [SITBI] | 2.26 [1.96, 2.59] | 2.28 [1.91, 2.73] | 2.15 [1.87, 2.47] |
| Psychological openness [IASMHS] | 1.06 [1.00, 1.12] | 1.22 [1.12, 1.33] | 1.05 [0.99, 1.12] |
| Help-seeking propensity [IASMHS] | 1.50 [1.40, 1.59] | 2.21 [2.00, 2.44] | 1.45 [1.36, 1.54] |
| Indifference to stigma [IASMHS] | 1.20 [1.13, 1.27] | 0.97 [0.89, 1.05] | 1.21 [1.14, 1.28] |
| Barriers for help-seeking Behavior | 1.04 [0.98, 1.10] | 0.70 [0.64, 0.77] | 1.07 [1.01, 1.13] |
| Clinical group-status [yes] | 0.75 [0.63, 0.90] | 0.75 [0.59, 0.95] | 0.75 [0.63, 0.89] |
| Total explained pseudo R^2^ | 0.20 | 0.23 | 0.19 |

*Notes: AHSQ* Actual Help-Seeking Questionnaire, *AUDIT* Alcohol Use Disorders Identification Test*, ChEDE-Q* Eating Disorder Examination-Questionnaire for children, *FAS* Family Affluence Scale, *IASMHS* Inventory of Attitudes Towards Seeking Mental Health Services, *NSSI* non-suicidal self-injury, *PHQ-A* Patient Health Questionnaire-9 modified for adolescents, *SAPAS* Self-Rated Standardized Assessment of Personality - Abbreviated Scale, *SDQ* Strengths and Difficulties Questionnaire, *SITBI* Self-Injurious Thoughts and Behavior Interview

Additional analyses using the SDQ subscales as predictors

As suggested by a reviewer, we repeated the regression analyses predicting help-seeking attitude (IASMHS subscales) and behavior (AHSQ subscales) using the SDQ subscales (i.e., conduct problems, emotional symptoms, hyperactivity/inattention, peer relationship problems, and prosocial behavior), the KIDSCREEN-10 and the PHQ-9 as individual predictors instead of the general psychopathology composite score. The results are presented in Tables 3 -6.

Notably, we decided to conduct linear regression analyses for both the three IASMHS subscales and the three AHSQ subscales, despite the latter being dichotomous (actual, professional, and informal help-seeking: yes/no). This decision was made because the computational time increases significantly with each additional predictor when applying the LGM formula. Importantly, the type of regression analysis (linear versus logistic) does not significantly influence the relative importance of predictors as estimated by the LGM formula. For illustration purposes, we repeated the models predicting the AHSQ subscales by the general psychopathology composite score with linear regression instead of logistic regression, as presented in Table 3 of the main manuscript. The results of the linear regression analyses are shown in Table 7. Notably, the relative importance of predictors based on linear regression is comparable to that based on logistic regression. This supports our approach of switching from logistic to linear regression for our secondary analyses including the SDQ subscales as predictors.

*Table 3.* Respective proportions (in %) of the total variance (R^2^) of psychological openness, help-seeking propensity, and indifference to stigma (IASMHS) explained by each predictor, including the SDQ subscales.

| Predictor variables | Psychological openness (IASMHS) | Help-seeking propensity (IASMHS) | Indifference to stigma (IASMHS) |
| --- | --- | --- | --- |
| Age | 0.26 | 0.10 | 1.99 |
| Sex | 0.17 | 0.58 | 0.17 |
| Center | 0.51 | 0.53 | 0.13 |
| Migration status | 0.21 | 0.86 | 0.13 |
| Socioeconomic status [FAS] | 0.02 | 0.85 | 0.04 |
| Psychosocial adversity [Laucht-Index] | 0.17 | 0.25 | 0.16 |
| Health-related quality of life [Kidscreen-10] | 0.32 | 1.36 | 1.41 |
| Severity of depressive symptoms [PHQ-A] | 1.11 | 0.76 | 1.82 |
| Conduct problems [SDQ] | 0.21 | 1.77 | 0.52 |
| Emotional symptoms [SDQ] | 0.24 | 0.22 | 1.00 |
| Hyperactivity/inattention [SDQ] | 0.13 | 0.67 | 0.33 |
| Peer relationship problems [SDQ] | 0.57 | 0.80 | 1.21 |
| Prosocial behavior [SDQ] | 0.30 | 1.89 | 0.73 |
| Eating disorder pathology [ChEDE-Q] | 1.03 | 0.36 | 3.40 |
| Personality pathology (SAPAS] | 0.82 | 0.14 | 1.04 |
| Alcohol problems [AUDIT] | 0.19 | 0.10 | 0.09 |
| Suicidal ideation in the last 12 months [Paykel] | 0.79 | 0.30 | 0.89 |
| Suicide attempt in the last 12 months [Paykel] | 0.04 | 0.13 | 0.30 |
| Lifetime NSSI [SITBI] | 0.21 | 0.16 | 0.30 |
| Clinical group status | 0.26 | 0.35 | 0.86 |
| Total explained variance (R^2^) | 7.55 | 12.16 | 16.53 |

*Notes: AUDIT* Alcohol Use Disorders Identification Test*, ChEDE-Q* Eating Disorder Examination-Questionnaire for children, *FAS* Family Affluence Scale, *IASMHS* Inventory of Attitudes toward Seeking Mental Health Services, *LMG* Formula of Lindeman, Merenda and Gold, *NSSI* non-suicidal self-injury, *PHQ-A* Patient Health Questionnaire-9 modified for adolescents, *SAPAS* Self-Rated Standardized Assessment of Personality - Abbreviated Scale, *SDQ* Strengths and Difficulties Questionnaire, *SEED* Short Evaluation of Eating Disorder, *SITBI* Self-Injurious Thoughts and Behavior Interview

*Table 4.* Regression parameters [95% Confidence Interval] for the predictors of psychological openness, help-seeking propensity, and indifference to stigma (IASMHS subscales), including the SDQ subscales.

| Predictor variables | Psychological  openness (IASMHS) | Help-seeking  propensity (IASMHS) | Indifference to stigma (IASMHS) |
| --- | --- | --- | --- |
| Age | 0.39 [0.27, 0.51] | 0.23 [0.09, 0.38] | 0.91 [0.79, 1.03] |
| Sex [female] | 0.51 [0.27, 0.75] | 0.96 [0.67, 1.24] | 0.60 [0.36, 0.84] |
| Center [Hamburg] | -0.45 [-0.76, -0.15] | 0.59 [0.22, 0.95] | -0.04 [-0.35, 0.26] |
| Center [Leipzig] | -1.08 [-1.38, -0.79] | 0.57 [0.23, 0.92] | 0.23 [-0.06, 0.52] |
| Center [Schwäbisch Gmünd] | -0.53 [-0.82, -0.23] | -0.04 [-0.39, 0.31] | -0.15 [-0.44, 0.15] |
| Center [Marburg] | -0.36 [-0.75, 0.03] | 1.33 [0.87, 1.79] | -0.17 [-0.55, 0.22] |
| Migration background [yes] | -0.52 [-0.76, -0.29] | -0.94 [-1.23, -0.66] | -0.29 [-0.53, -0.05] |
| Migration status unknown [yes] | 0.37 [-0.31, 1.06] | -1.31 [-2.12, -0.50] | -0.22 [-0.90, 0.46] |
| Socioeconomic status [FAS] | -0.06 [-0.18, 0.06] | 0.48 [0.34, 0.61] | 0.05 [-0.07, 0.17] |
| Psychosocial adversity [Laucht-Index] | -0.06 [-0.13, 0.02] | 0.03 [-0.06, 0.11] | 0.17 [0.09, 0.24] |
| Health-related quality of life [Kidscreen-10] | -0.03 [-0.05, 0.00] | 0.12 [0.09, 0.15] | 0.07 [0.04, 0.09] |
| Severity of depressive symptoms [PHQ-A] | -0.14 [-0.18, -0.10] | -0.03 [-0.08, 0.02] | -0.06 [-0.09, -0.02] |
| Conduct problems [SDQ] | -0.03 [-0.16, 0.09] | -0.58 [-0.73, -0.44] | -0.10 [-0.22, 0.02] |
| Emotional symptoms [SDQ] | 0.20 [0.03, 0.37] | 0.08 [-0.12, 0.28] | -0.17 [-0.34, -0.00] |
| Hyperactivity/inattention [SDQ] | 0.01 [-0.11, 0.13] | -0.25 [-0.39, -0.11] | 0.00 [-0.11, 0.12] |
| Peer relationship problems [SDQ] | -0.32 [-0.44, -0.20] | -0.36 [-0.50, -0.22] | -0.41 [-0.53, -0.29] |
| Prosocial behavior [SDQ] | 0.19 [0.08, 0.31] | 0.68 [0.55, 0.82] | 0.35 [0.24, 0.46] |
| Eating disorder pathology [ChEDE-Q] | -0.48 [-0.61, -0.35] | -0.29 [-0.44, -0.14] | -1.01 [-1.13, -0.88] |
| Personality pathology [SAPAS] | -0.42 [-0.54, -0.29] | 0.20 [0.05, 0.34] | -0.37 [-0.49, -0.24] |
| Alcohol problems [AUDIT] | -0.28 [-0.40, -0.16] | -0.18 [-0.33, -0.04] | -0.10 [-0.22, 0.02] |
| Suicidal ideation in the last 12 months [Paykel] | -0.78 [-1.04, -0.51] | -0.27 [-0.58, 0.05] | -0.50 [-0.76, -0.23] |
| Suicide attempt in the last 12 months [Paykel] | 0.57 [-0.07, 1.21] | -0.48 [-1.24, 0.27] | -0.73 [-1.37, -0.10] |
| Lifetime NSSI [SITBI] | -0.14 [-0.45, 0.18] | -0.08 [-0.46, 0.30] | 0.28 [-0.03, 0.60] |
| Clinical group-status [yes] | 1.02 [0.62, 1.41] | 0.46 [-0.01, 0.93] | -0.07 [-0.46, 0.33] |
| Total explained variance (R^2^) | 0.08 | 0.12 | 0.17 |

*Notes: AUDIT* Alcohol Use Disorders Identification Test*, ChEDE-Q* Eating Disorder Examination-Questionnaire for children, *FAS* Family Affluence Scale, *IASMHS* Inventory of Attitudes Towards Seeking Mental Health Services, *NSSI* non-suicidal self-injury, *PHQ-A* Patient Health Questionnaire-9 modified for adolescents, *SAPAS* Self-Rated Standardized Assessment of Personality - Abbreviated Scale, *SDQ* Strengths and Difficulties Questionnaire, *SITBI* Self-Injurious Thoughts and Behavior Interview

*Table 5.* Respective proportions (in %) of the total variance (R^2^) of actual help-seeking, professional help-seeking, and informal help-seeking (AHSQ) explained by each predictor, including the SDQ subscales.

| Predictor variables | Actual help-seeking (AHSQ) | Professional help-seeking (AHSQ) | Informal help-seeking (AHSQ) |
| --- | --- | --- | --- |
| Age | 1.51 | 0.45 | 1.53 |
| Sex | 0.72 | 0.30 | 0.71 |
| Center | 0.30 | 0.13 | 0.26 |
| Migration status | 0.26 | 0.16 | 0.23 |
| Socioeconomic status [FAS] | 0.15 | 0.06 | 0.16 |
| Psychosocial adversity [Laucht-Index] | 0.75 | 0.73 | 0.64 |
| Health-related quality of life [Kidscreen-10] | 2.30 | 1.15 | 2.03 |
| Severity of depressive symptoms [PHQ-A] | 2.61 | 1.21 | 2.30 |
| Conduct problems [SDQ] | 0.10 | 0.07 | 0.09 |
| Emotional symptoms [SDQ] | 4.21 | 1.89 | 3.96 |
| Hyperactivity/inattention [SDQ] | 0.15 | 0.09 | 0.13 |
| Peer relationship problems [SDQ] | 0.37 | 0.49 | 0.29 |
| Prosocial behavior [SDQ] | 0.24 | 0.07 | 0.31 |
| Eating disorder pathology [ChEDE-Q] | 0.82 | 0.37 | 0.71 |
| Personality pathology [SAPAS] | 1.08 | 0.73 | 1.00 |
| Alcohol problems [AUDIT] | 0.87 | 0.27 | 0.89 |
| Suicidal ideation in the last 12 months [Paykel] | 2.27 | 0.81 | 1.98 |
| Suicide attempt in the last 12 months [Paykel] | 0.29 | 0.67 | 0.18 |
| Lifetime NSSI [SITBI] | 3.25 | 2.12 | 2.97 |
| Psychological openness [IASMHS] | 0.08 | 0.51 | 0.08 |
| Help-seeking propensity [IASMHS] | 1.09 | 2.82 | 0.92 |
| Indifference to stigma [IASMHS] | 0.48 | 0.17 | 0.51 |
| Barriers to help-seeking behavior | 0.13 | 1.52 | 0.13 |
| Clinical group status | 0.93 | 0.61 | 0.80 |
| Total explained variance (R^2^) | 24.98 | 17.40 | 22.85 |

*Notes: AHSQ* Actual Help-Seeking Questionnaire, *AUDIT* Alcohol Use Disorders Identification Test, *ChEDE-Q* Eating Disorder Examination-Questionnaire for children, *FAS* Family Affluence Scale, *IASMHS* Inventory of Attitudes Towards Seeking Mental Health Services, *LMG* Formula of Lindeman, Merenda and Gold, *PHQ-A* PHQ-9 modified for adolescents, *SAPAS* Self-Rated Standardized Assessment of Personality - Abbreviated Scale, *SDQ* Strengths and Difficulties Questionnaire, *SEED* Short Evaluation of Eating Disorder, *SITBI* Self-Injurious Thoughts and Behavior Interview

*Table 6.* Regression parameters [95% Confidence Interval] for the predictors of actual help-seeking, professional help-seeking and informal help-seeking (AHSQ subscales), including the SDQ subscales.

| Predictor variables | Actual help-  seeking (AHSQ) | Professional help-seeking (AHSQ) | Informal help-seeking (AHSQ) |
| --- | --- | --- | --- |
| Age | 1.28 [1.20, 1.36] | 1.15 [1.06, 1.25] | 1.28 [1.21, 1.36] |
| Sex [female] | 1.18 [1.04, 1.33] | 1.19 [0.99, 1.41] | 1.19 [1.05, 1.35] |
| Center [Hamburg] | 1.17 [1.00, 1.37] | 1.03 [0.83, 1.27] | 1.15 [0.99, 1.35] |
| Center [Leipzig] | 1.19 [1.03, 1.38] | 0.97 [0.79, 1.18] | 1.16 [1.00, 1.35] |
| Center [Schwäbisch Gmünd] | 0.93 [0.80, 1.08] | 0.84 [0.67, 1.05] | 0.93 [0.80, 1.09] |
| Center [Marburg] | 0.98 [0.81, 1.19] | 1.04 [0.81, 1.34] | 0.97 [0.80, 1.17] |
| Migration background [yes] | 0.78 [0.69, 0.88] | 0.73 [0.62, 0.87] | 0.81 [0.72, 0.92] |
| Migration status unknown [yes] | 0.74 [0.52, 1.06] | 0.99 [0.62, 1.58] | 0.73 [0.51, 1.05] |
| Socioeconomic status [FAS] | 1.16 [1.09, 1.23] | 1.10 [1.02, 1.20] | 1.17 [1.10, 1.24] |
| Psychosocial adversity [Laucht-Index] | 1.10 [1.06, 1.14] | 1.15 [1.10, 1.21] | 1.09 [1.05, 1.13] |
| Health-related quality of life [Kidscreen-10] | 0.97 [0.96, 0.99] | 0.97 [0.95, 0.99] | 0.98 [0.96, 0.99] |
| Severity of depressive symptoms [PHQ-A] | 1.04 [1.02, 1.06] | 1.02 [0.99, 1.04] | 1.03 [1.02, 1.05] |
| Conduct problems [SDQ] | 0.99 [0.93, 1.06] | 1.06 [0.97, 1.15] | 0.99 [0.93, 1.05] |
| Emotional symptoms [SDQ] | 1.62 [1.49, 1.76] | 1.50 [1.34, 1.68] | 1.61 [1.48, 1.75] |
| Hyperactivity/inattention [SDQ] | 0.94 [0.88, 1.00] | 1.01 [0.93, 1.10] | 0.95 [0.89, 1.01] |
| Peer relationship problems [SDQ] | 1.03 [0.97, 1.10] | 1.18 [1.09, 1.28] | 1.01 [0.95, 1.08] |
| Prosocial behavior [SDQ] | 1.09 [1.03, 1.16] | 1.04 [0.96, 1.13] | 1.11 [1.05, 1.18] |
| Eating disorder pathology [ChEDE-Q] | 0.99 [0.93, 1.06] | 0.99 [0.92, 1.07] | 0.99 [0.93, 1.05] |
| Personality pathology [SAPAS] | 1.13 [1.06, 1.20] | 1.19 [1.10, 1.30] | 1.13 [1.06, 1.20] |
| Alcohol problems [AUDIT] | 1.15 [1.09, 1.22] | 1.11 [1.04, 1.20] | 1.15 [1.09, 1.22] |
| Suicidal ideation in the last 12 months [Paykel] | 1.64 [1.45, 1.86] | 1.46 [1.22, 1.74] | 1.59 [1.40, 1.80] |
| Suicide attempt in the last 12 months [Paykel] | 1.44 [1.06, 1.95] | 2.12 [1.53, 2.93] | 1.17 [0.87, 1.58] |
| Lifetime NSSI [SITBI] | 2.25 [1.95, 2.59] | 2.27 [1.90, 2.72] | 2.15 [1.86, 2.47] |
| Psychological openness [IASMHS] | 1.04 [0.98, 1.10] | 1.20 [1.10, 1.31] | 1.03 [0.97, 1.10] |
| Help-seeking propensity [IASMHS] | 1.43 [1.34, 1.53] | 2.16 [1.96, 2.39] | 1.38 [1.29, 1.47] |
| Indifference to stigma [IASMHS] | 1.21 [1.14, 1.29] | 0.98 [0.90, 1.06] | 1.22 [1.15, 1.29] |
| Barriers for help-seeking Behavior | 1.01 [0.95, 1.07] | 0.70 [0.64, 0.76] | 1.04 [0.98, 1.10] |
| Clinical group-status [yes] | 0.77 [0.64, 0.92] | 0.79 [0.62, 1.01] | 0.77 [0.64, 0.92] |
| Total explained variance (R^2^) | 0.22 | 0.23 | 0.20 |

*Notes: AHSQ* Actual Help-Seeking Questionnaire, *AUDIT* Alcohol Use Disorders Identification Test*, ChEDE-Q* Eating Disorder Examination-Questionnaire for children, *FAS* Family Affluence Scale, *IASMHS* Inventory of Attitudes Towards Seeking Mental Health Services, *NSSI* non-suicidal self-injury, *PHQ-A* Patient Health Questionnaire-9 modified for adolescents, *SAPAS* Self-Rated Standardized Assessment of Personality - Abbreviated Scale, *SDQ* Strengths and Difficulties Questionnaire, *SITBI* Self-Injurious Thoughts and Behavior Interview

***Table 7.*** Respective proportions (in %) of the total variance (R2) of actual help-seeking, professional help-seeking, and informal help-seeking (AHSQ) explained by each predictor.

| Predictor variables | Actual help-seeking (AHSQ) | Professional help-seeking (AHSQ) | Informal help-seeking (AHSQ) |
| --- | --- | --- | --- |
| Age | 7.91 | 3.31 | 8.85 |
| Sex | 4.99 | 2.65 | 5.50 |
| Center | 1.29 | 0.87 | 1.24 |
| Migration status | 1.22 | 1.00 | 1.20 |
| Socioeconomic status [FAS] | 0.65 | 0.40 | 0.78 |
| Psychosocial adversity [Laucht-Index] | 3.96 | 5.20 | 3.69 |
| General psychopathology [SDQ, Kidscreen-10, PHQ-A] | 20.43 | 16.32 | 19.73 |
| Eating disorder pathology [ChEDE-Q] | 4.97 | 3.17 | 4.79 |
| Personality pathology [SAPAS] | 6.56 | 6.23 | 6.63 |
| Alcohol problems [AUDIT] | 3.78 | 1.64 | 4.23 |
| Suicidal ideation in the last 12 months [Paykel] | 12.35 | 6.40 | 11.88 |
| Suicide attempt in the last 12 months [Paykel] | 1.53 | 4.34 | 1.09 |
| Lifetime NSSI [SITBI] | 16.23 | 14.36 | 16.32 |
| Psychological openness [IASMHS] | 0.42 | 2.97 | 0.44 |
| Help-seeking propensity [IASMHS] | 5.27 | 16.78 | 5.04 |
| Indifference to stigma [IASMHS] | 1.82 | 0.85 | 2.16 |
| Barriers to help-seeking behavior | 0.71 | 7.96 | 0.86 |
| Clinical group status | 5.92 | 5.56 | 5.56 |
| Total explained variance (R^2^) | 23.55 | 16.98 | 21.37 |

*Notes: AHSQ* Actual Help-Seeking Questionnaire, *AUDIT* Alcohol Use Disorders Identification Test, *ChEDE-Q* Eating Disorder Examination-Questionnaire for children, *FAS* Family Affluence Scale, *IASMHS* Inventory of Attitudes Towards Seeking Mental Health Services, *LMG* Formula of Lindeman, Merenda and Gold, *PHQ-A* PHQ-9 modified for adolescents, *SAPAS* Self-Rated Standardized Assessment of Personality - Abbreviated Scale, *SDQ* Strengths and Difficulties Questionnaire, *SEED* Short Evaluation of Eating Disorder, *SITBI* Self-Injurious Thoughts and Behavior Interview

References

1. the ProHEAD Consortium, Kaess M, Ritter S, et al (2019) Promoting Help-seeking using E-technology for ADolescents with mental health problems: study protocol for a randomized controlled trial within the ProHEAD Consortium. Trials 20:94. https://doi.org/10.1186/s13063-018-3157-7

2. Bauer S, Winn S, Schmidt U, Kordy H (2005) Construction, scoring and validation of the Short Evaluation of Eating Disorders (SEED). Eur Eat Disord Rev 13:191–200. https://doi.org/10.1002/erv.637

3. Johnson JG, Harris ES, Spitzer RL, Williams JBW (2002) The patient health questionnaire for adolescents. J Adolesc Health 30:196–204. https://doi.org/10.1016/S1054-139X(01)00333-0

4. Babor TF, Higgins-Biddle JC, Saunders JB, Monteiro MG (2001) AUDIT: the alcohol use disorders identification test: Guidelines for use in primary health care. World Health Organization

5. Goodman A, Goodman R (2009) Strengths and Difficulties Questionnaire as a Dimensional Measure of Child Mental Health. J Am Acad Child Adolesc Psychiatry 48:400–403. https://doi.org/10.1097/CHI.0b013e3181985068
